# Interleukin-18 Is a Prognostic Marker and Plays a Tumor Suppressive Role in Colon Cancer

**DOI:** 10.1155/2020/6439614

**Published:** 2020-11-26

**Authors:** Xiaodong Feng, Zhijun Zhang, Peng Sun, Guanghui Song, Lu Wang, Zhenqing Sun, Ning Yuan, Qing Wang, Limin Lun

**Affiliations:** ^1^Department of Clinical Laboratory, The Affiliated Hospital of Qingdao University, Qingdao University, Qingdao 266000, China; ^2^Department of Clinical Laboratory, Taian City Central Hospital, Taian 271000, China; ^3^Department of Pathology, Taian City Central Hospital, Taian 271000, China; ^4^Department of Education and Training, The Affiliated Hospital of Qingdao University, Qingdao University, Qingdao 266000, China; ^5^Department of General Surgery, The Affiliated Hospital of Qingdao University, Qingdao University, Qingdao 266000, China; ^6^Department of Clinical Laboratory, The Seventh Medical Center of PLA General Hospital, Bejing 100010, China

## Abstract

Interleukin-18 (IL-18) belongs to the IL-1 family and is an essential proinflammatory and immune regulatory cytokine. The present study was designed to investigate the expression and function of IL-18 in colon cancer. In clinical analyses, mRNA and protein expressions of IL-18 were decreased in tissues of colon cancer patients. This decreased expression of IL-18 was significantly correlated with the tumor size (*P* = 0.001) and American Joint Committee on Cancer (AJCC) stage (*P* = 0.013). Patients with IL-18-positive tumors had a better survival rate than patients with IL-18-negative tumors. Moreover, upregulation of IL-18 inhibited colon cancer cell proliferation. Our data suggest that the decreased expression of IL-18 in colon cancer was associated with prognosis and tumor proliferation. IL-18 may be considered a novel tumor suppressor and a potential therapeutic target for colon cancer patients.

## 1. Introduction

Colon cancer is a collective group of cancers that involve the colon, the rectum, and the appendix. As the most common malignant tumor, it takes up approximately 6.1% of annual cancer incidence worldwide [1]. Although many new therapeutic improvements have been proposed for treating colon cancer, the long-term survival rate is unsatisfactory due to tumor recurrence and metastasis, and the 5-year survival rate remains only approximately 50% [2]. To improve the treatment outcomes of colon cancer patients, a comprehensive study of the molecular mechanisms underlying colon cancer progression is needed.

The interleukin-1 (IL-1) family comprises 11 members, including seven proinflammatory cytokines and one anti-inflammatory cytokine [3]. Evidences have demonstrated that they are key regulators of multiple physiological and pathological processes, including innate immune and inflammatory responses [4, 5]. Among the IL-1 family, interleukin-18 (IL-18) is one of the best characterized. The protein encoded by the IL-18 gene, located at 11q23.1, is essential for the response to the pathogens and activation of host defense mechanisms [6]. IL-18 has been shown to be a mediate product of activation by NOD-like receptor pyrin domain-containing protein 3 (NLRP3) inflammasome/caspase-1 signaling pathway [7]. It is secreted mainly by macrophages and dendritic cells and stimulates interferon-*γ* (IFN-*γ*) production by natural killer (NK) cells and thymus-dependent lymphocyte (T cells) [8]. IL-18 is not only the critical regulators of signaling pathways in a variety of immune responses but also involved in antitumor process in a variety of cancers. Recently, aberrant low expression of IL-18 has been identified in some digestive system cancers, such as esophageal cancer and oral squamous cell carcinoma [9, 10]. Lebel-Binay et al. also proved that high IL-18 expression in prostate cancer tissues might be prognostic for better outcome of patients, independently of clinicopathologic features [11]. However, the expression and the role of IL-18 in colon cancer tissue remain less understood.

Here, we explored the expression of IL-18 in colon cancer tissues and analyzed the correlation between the expression and the prognosis of patients. We found that the expression of IL-18 was significantly decreased in colon cancer tissues. By upregulating the expression of IL-18 with plasmid, we investigated the functional relevance of IL-18 on cellular proliferation in colon cancer cell. Our findings imply that IL-18 is implicated as a potential prognostic indicator and therapeutic target in colon cancer.

## 2. Methods

### 2.1. Patients

The retrospective study included 116 colon cancer patients admitted to the Affiliated Hospital of Qingdao University between 2013 and 2014. All cases were histologically confirmed by two pathologists, and diagnoses and pathological staging were determined in accordance with the American Joint Committee on Cancer (AJCC). The study was approved by the Independent Ethics Committee of the Affiliated Hospital of Qingdao University, and all of the specimens were collected with the patients' consent.

### 2.2. Cell Line

The colon cancer cell line RKO was obtained from the Cell Resource Center of Shanghai Institutes for Biological Sciences, Type Culture Collection of the Chinese Academy of Sciences (Shanghai, China). The cell line was maintained in Dulbecco's Modified Eagle Media (DMEM) supplemented with 10% fetal bovine serum (FBS) (Gibco, USA) in a humidified atmosphere of 5% CO_2_ at 37°C.

### 2.3. Analysis of IL-18 Expression in Published Datasets

The Cancer Genome Atlas (TCGA) data were obtained from the web portal (https://tcga-data.nci.nih.gov/tcga/). The TCGA dataset used in this study comprised messenger ribonucleic acid (mRNA) expression data from 41 normal and 286 colon adenocarcinoma (COAD) primary tumor samples, respectively. Gene expression data from the early-onset colorectal cancer (CRC) was processed by the ScanGEO (http://scangeo.dartmouth.edu/ScanGEO/) [12]. The dataset used in this study comprised expression profiling of 22 samples (GEO; GDS2609). Raw data is available from the Gene Expression Omnibus (GEO). The interactive survival scatter plot and survival analysis from 597 TCGA RNA samples were analyzed by the Human Protein Atlas (https://www.proteinatlas.org/).

### 2.4. Quantitative Real-Time Polymerase Chain Reaction (PCR) (qPCR)

Total RNA was isolated with TRIzol (TaKaRa, Shiga, Japan) following the manufacturer's instructions, and complementary deoxyribonucleic acid (cDNA) was synthesized using the PrimeScript RT Reagent kit (TaKaRa). The mRNA transcripts were analyzed by Applied Biosystems 7900 (ABI 7900) Real-time PCR System (ABI, Foster City, USA) with the SYBR Premix Ex Taq II (TaKaRa) reaction system. All relative quantifications were calculated by normalizing with the glyceraldehyde-3-phosphate dehydrogenase (GAPDH) expression. The primer sequences were as follows: GAPDH: sense 5′- AGAAGGCTGGGGCTCATTTG -3′, antisense 5′- AGGGGCCATCCACAGTCTTC -3′ and IL-18: sense 5′-CGTAGTCGGATGACCCTTCT-3′, antisense 5′-ACTTCTCGATCGCCGGATA-3′.

### 2.5. Western Blot

Total protein was extracted from colon cancer tissues or paired normal tissues with radioimmunoprecipitation assay (RIPA) lysis buffer (Thermo Scientific, Rockford, IL, USA). Equal amounts of total protein were electrophoresed in a 10% sodium dodecyl sulfate- (SDS-) polyacrylamide gels, transferred onto polyvinylidene fluoride (PVDF) membranes (Millipore, Shanghai, China), and blocked in 5% nonfat dry milk for 60 min. Then, the membranes were incubated with the following primary antibodies overnight at 4°C: *β*-actin (1 : 1000, Sigma) and IL-18 (1 : 800, CST, Danvers, MA, USA). Followed by incubation with a secondary antibody, the protein was visualized by the enhanced chemiluminescence (ECL) detection system (Pierce Biotechnology, Rockford, IL, USA).

### 2.6. Immunohistochemistry (IHC) Staining

IHC was performed as previously described [13]. Immunostaining was conducted using the primary antibodies against IL-18 (1 : 200, Cell Signaling Technology (CST)), Ki-67 (1 : 400, CST), and cleaved caspase-3 (1 : 400, CST). Then, the tissue sections were incubated with horseradish peroxidase- (HRP-) conjugated secondary antibody (GeneTech, Shanghai, China). For IL-18 and cleaved caspase-3, the staining intensity (SI) was scored as zero (negative), one (weak), two (moderate), or three (strong) and the staining extent (SE) was scored as zero (0%), one (1%–25%), two (26%–50%), three (51%–75%), or four (76%–100%). The overall staining score (OSC) was calculated by the product of the SI score and SE score. According to the OSC, specimens were divided into different groups as follows: the negative expression (0–4), weak expression (5–8), and strong expression (9–12) groups. For Ki-67, patients were also grouped by different expression: the low-expression group (nuclear staining tumor cells < 46%) and high-expression group (nuclear staining tumor cells ≥ 46%).

### 2.7. Transfection of IL-18 in RKO Cell Line

The GV144 plasmid coding for IL-18 was purchased from GeneChem (Shanghai, China). For transient transfection, 5 × 10^4^ RKO cells in 6-well plates were cultured to 75% confluence. And then, the cells were transfected with GV144-IL18 or empty vector controls using Lipofectamine 2000 (Invitrogen). The expression of IL-18 was confirmed by real-time PCR and western blot analysis.

### 2.8. Cell Counting Kit-8 (CCK-8) Assays and Colony Formation Assays

For CCK-8 assays, 1 × 10^4^ RKO cells were plated in 96-well plates and cultured for 24, 48, 72, 96, or 120 h. Then, 8 *μ*l CCK-8 (Dojindo, Tokyo, Japan) was added to each well for additional 2 h incubation. Finally, optical density values at 450 nm were measured with a microplate reader (Bio-Rad, Hercules, CA, USA). For plate colony formation assays, 5 × 10^2^ cells were plated in 6-well plates and cultured for 12 days. After fixing in paraformaldehyde for 30 min, colonies were stained with Giemsa stain solution for 20 min and counted.

### 2.9. Statistical Analysis

The statistical significance of differences between two groups was determined by two-tailed Student's *t*-test. The relationship of IL-18 expression with clinicopathological parameters were analyzed using chi-squared tests. The correlation between IL-18 and Ki-67 and cleaved caspase-3 protein expressions was analyzed using Spearman's correlation coefficient test. Overall survival (OS) rates and disease-free survival (DFS) rates were calculated by the Kaplan–Meier method. A log-rank test was used to compare the survival curves. *P* < 0.05 was considered statistically significant. All statistical analyses were carried out by Statistical Product and Service Solutions (SPSS) 19.0 (SPSS, Inc., Chicago, IL).

## 3. Result

### 3.1. Expression of IL-18 in Colon Cancer Tissues

**Figure 1:**
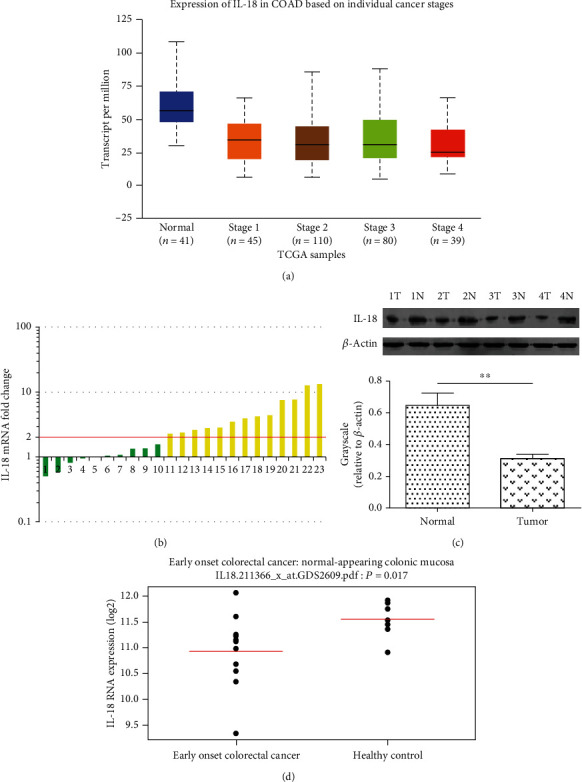
Expression of IL-18 in human colon cancer tissues. (a) The mRNA expression of IL-18 in 41 normal and 274 different grades of colon cancer. The data and *P* values were obtained from the TCGA database. (b) The mRNA expression levels of IL-18 in 23 paired colon cancer and adjacent normal tissues were determined by real-time qPCR. (c) Western blot analysis of IL-18 protein in 4 paired colon cancer and adjacent normal tissues. (d) Expression levels of IL-18 in early onset colorectal cancer and healthy control in the Hong clinical cohort. ^∗^*P* < 0.05, ^∗∗^*P* < 0.01.

To explore the possible roles of IL-18 in development of colon cancer, we first analyzed the TCGA database and found that IL-18 was significantly lower in colon cancer tissues than normal tissues, and this downregulated expression was happened on the early stage of the disease (Figure 1(a)). Among 23 paired colon cancer cases that were assessed for expression of IL-18 mRNA, 13 (56.5%, *P* < 0.01) adjacent noncancerous tissues showed a more than 2-fold increase compared with tumor tissues (Figure 1(b)). Likewise, western blot analyses confirmed a significant downregulation of IL-18 protein in colon cancer tissues compared with adjacent noncancerous tissues (Figure 1(c)). In the Hong clinical cohort, the expression of IL-18 was decreased in early-onset colorectal cancer samples compared with healthy control samples (*P* = 0.001, Figure 1(d)). These results demonstrate that the IL-18 expression is commonly reduced in human colon cancer tissues.

### 3.2. Correlation between IL-18 Expression and Colon Cancer Clinicopathologic Parameters

**Figure 2:**
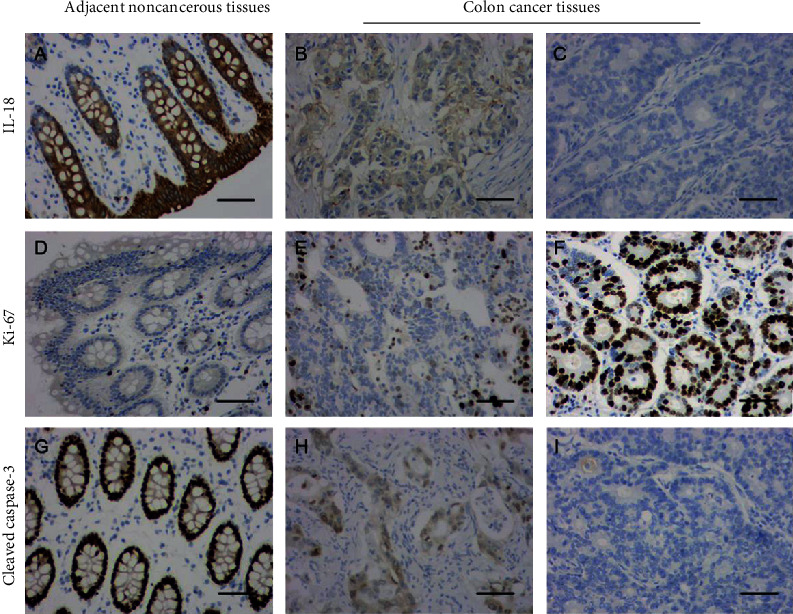
Immunohistochemical staining of IL-18, Ki-67, and cleaved caspase-3 expression in colon cancer. The expression of IL-18 (a–c), Ki-67 (d–f), and cleaved caspase-3 (g–i) in colon cancer tissues and adjacent noncancerous tissues was examined by immunohistochemical staining. Original magnification ×200 (scale bars =100 *μ*m).

To explore the clinicopathologic significance of IL-18 expression, we used IHC to detect the expression of IL-18 in tissues from 116 cases of primary colon cancer. IL-18 staining was predominantly localized in the cytoplasm of colonic epithelial and tumor cells. Of the 116 colon cancer specimens, only 17 (14.7%) showed strong IL-18 expression, while 76 (65.5%) showed negative IL-18 expression (Figures 2(a)–2(c), Table 1. The associations of clinicopathologic factors and IL-18 expression are shown in Table 1 Downregulation of IL-18 was associated with T stage (*P* = 0.001) and AJCC stage (*P* = 0.013) (Table 1). To further investigate the relationship between IL-18 status with proliferation and apoptosis, we examine the expression of Ki-67 and cleaved caspase-3 in the tissues. The results showed that the expression of Ki-67 was increased in the colon cancer tissues, while the expression of cleaved caspase-3 was reduced (Figures 2(d)–2(i)). Moreover, a negative correlation between Ki-67 and IL-18 was identified by Spearman's correlation coefficient test (*P* = 0.003, Table 2). However, the result indicated no significant correlation of cleaved caspase-3 with the IL-18 expression in colon cancer patients (*P* = 0.07, Table 2). Above results indicate that IL-18 is correlated with colon cancer growth and progression.

### 3.3. Low IL-18 Expression Predicts Poor Clinical Outcomes of Colon Cancer

**Figure 3:**
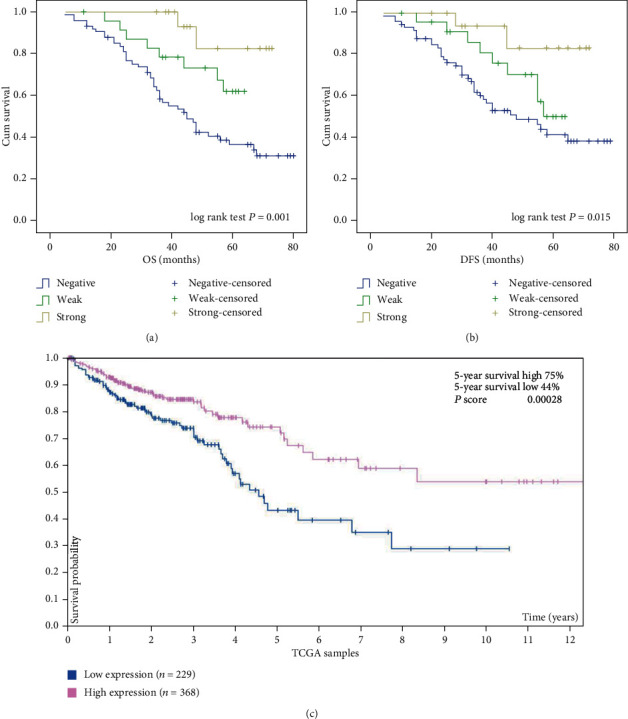
Kaplan–Meier with log rank test analysis of the OS rate and DFS rate for colon cancer patients. OS rate (a) and DFS rate (b) of patients in relation to their expression levels of IL-18, as determined by immunohistochemical staining of tissue sections. (c) Kaplan–Meier analysis showing estimated overall survival rate in patients with high or low levels of IL-18 in the TCGA cohort.

We then assessed the association between the OS rates and DFS rates of colon cancer patients and IL-18 expression. Kaplan–Meier analysis showed that patients with low IL-18 expression had poorer OS and DFS rates (log rank test, *P* = 0.001 and *P* = 0.015, respectively) than patients with high levels of IL-18 (Figures 3(a) and 3(b)). In in silico analyses of TCGA colon cancer cohort, data also confirmed that patients with low IL-18 expression had a lower 10-year survival rate than patients with high IL-18 expression (*P* = 0.00028, Figure 3(c)). A Cox proportional hazard model demonstrated that low expression of IL-18 is a significant independent prognostic factor for decreased OS rate (hazard ratio (HR) 4.375, 95% confidence interval (CI) 1.279–8.220, *P* < 0.001; Table 3.

### 3.4. Overexpression of IL-18 Expression Suppresses Colon Cancer Cell Proliferation

**Figure 4:**
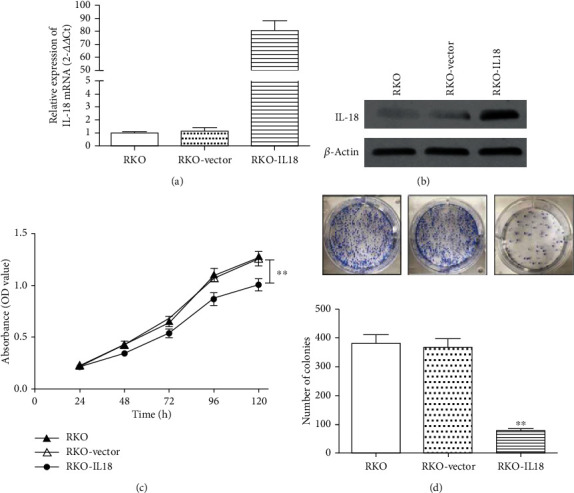
Effects of IL-18 on the proliferation of human colon cancer cells. RKO colon cancer cells were transfected with empty vector or plasmids coding for IL-18. The transfection efficiency was assessed by real-time PCR (a) and western blot (b). The effects of IL-18 overexpression on cell proliferation were evaluated with CCK-8 assays (c) and plate colony formation assays (d) (^∗^*P* < 0.05).

Taking these observations into account, we evaluated the effect of IL-18 on colon cancer cell proliferation. RKO cells were transfected with IL-18 expression or vector-control plasmids and analyzed. Real-time PCR and western blot analysis were employed to confirm that mRNA and protein levels of IL-18 were significantly increased in the overexpression cells (Figures 4(a) and 4(b)). CCK-8 assays demonstrated that overexpression of IL-18 expression significantly inhibited cell growth over time compared with control groups (*P* < 0.01, Figure 4(c)). Plate colony formation assays also suggested that IL-18 significantly inhibited cell clonogenicity compared with the control groups (*P* < 0.01, Figure 4(d)). These results indicate that IL-18 could suppress colon cancer cell proliferation.

## 4. Discussion

Colon cancer is the third leading cause of cancer-associated death in the world [14]. The disease develops from multiple alterations of gene expression; however, the related mechanisms remain unclear. Our study uncovers that IL-18 is involved in the progression and proliferation of colon cancer. The data revealed that low IL-18 expression in colon cancer tissues was associated with tumor size and AJCC stage and implicated that IL-18 is a prognostic factor for OS. Furthermore, our results indicate that IL-18 markedly represses colon cancer cell proliferation. However, the downstream mechanisms still require further investigation.

It is well accepted that proliferation is the important factor in tumor progression, and this progression depends on tumor cells acquiring the ability to growth [15]. Activation sustaining proliferative signaling has been considered as one of the hallmarks of cancer [16]. This activation is driven by activation of oncogenes and the inactivation of tumor suppressor genes as well as cytokine aberrations [17]. Cytokines are some small molecule proteins, which regulate multiple biological functions including innate and acquired immunity, hematopoiesis, and inflammation through mostly extracellular signaling [18, 19]. Cytokines have been known to be essential in embryogenesis and were demonstrated may play important roles in tumorigenesis and proliferation [20]. Among the large variety of cytokines, IL-18 is the better characterized one. IL-18 was once known as “IFN-*γ*-inducing factor,” owing to the ability to induce the expression of IFN-*γ* following treatment with lipopolysaccharide in mice [21]. Although dysfunction of IL-18 has been previously observed in inflammatory and autoimmune diseases, recent research has shown that IL-18 is also a key regulator of proliferation in some tumors [22]. To examine the role of IL-18 in protection against colorectal tumor development, Zaki et al. characterized colon inflammation and tumor development in *IL-18^−/−^* mice that were subjected to the azoxymethane (AOM)/dextran sodium sulfate (DSS) regimen. In agreement with an important role for IL-18 in protection against colitis-associated tumorigenesis, colons of *IL-18^−/−^* mice contained significantly more tumors than the treated wild-type mice. These results suggested that IL-18 signaling downstream of the NLRP3 inflammasome confers protection against colorectal tumorigenesis *in vivo*. [23]. Yang et al. evaluated synergistic antitumor activity of replication-competent adenovirus armed with IL-18 against melanoma and found that it synergistically inhibited the melanoma cells growth and inhibited the vascular endothelial growth factor expression [24]. Liu et al. have reported that introduction of the IL-18 gene inhibited human tongue squamous cell carcinoma cell line proliferation after transfection compared with the untransfected cells or cells transfected with blank pcDNA3.1(+) vector [25]. Xu et al. have shown that exogenous IL-18 significantly inhibited the proliferation and metastasis of esophageal squamous cell carcinoma cells [9]. However, the effects of IL-18 on CRC progression lie on multiple aspects. For example, Li et al. have demonstrated that the expression levels of IL-18 in the serum of patients with CRC were statistically higher than those of the control group. And the expressions of IL-18 of patients with reoccurred CRC after the operation were significantly higher than that of patients without recurrence of CRC in the study group [26]. Since IL-18 is produced by various hematopoietic and nonhematopoietic cells, including macrophages and dendritic cells [27]. More importantly, tumor-associated macrophages (TAMs) are important tumor-promoting cells in the CRC microenvironment [28, 29]. We speculate that the high level of IL-18 in the serum of CRC patient is mainly produced by the TAMs and associated with the poor prognosis and the shorter survival time. The IL-18 produced by different cells may explain in part the different expression and prognostic status of patients with CRC. Therefore, the underlying molecular mechanism responsible for the different functions of IL-18 in CRC required needs further study.

## 5. Conclusions

In this study, we confirmed that IL-18 expression was decreased in primary colon cancer tissues and this low expression was associated with T stage (*P* = 0.001) and AJCC stage (*P* = 0.013) as well as the OS rates. Moreover, we showed that colon cancer cellular proliferation may be potently dampened by the overexpression of IL-18 expression. Our study has identified that IL-18 may act as a colon cancer suppressor that inhibit the proliferation of colon cancer cells. These findings highlight the potential of IL-18 as a marker for patient prognosis and a therapeutic target in colon cancer.

## Figures and Tables

**Table 1:** Correlation between IL-18 expression and clinicopathological features in patients with colon cancer (*n* = 116).

Variable	*n*	IL-18 expression			*P* value^∗^
		Negative (76)	Weak (23)	Strong (17)	
Age					0.668
<60	54	36	9	9	
≥60	62	40	14	8	
Gender					0.826
Male	68	46	13	9	
Female	48	30	10	8	
Location					0.603
Right	58	38	13	7	
Transverse	29	17	7	5	
Left	29	21	3	5	
T stage					0.001^∗^
T1+T2	63	33	20	10	
T3+T4	53	43	3	7	
N stage					0.191
N0	64	46	9	9	
N1+N2	52	30	14	8	
M stage					0.585
M0	114	74	23	17	
M1	2	2	0	0	
AJCC stage					0.013^∗^
I	45	22	11	12	
II	22	12	7	3	
III	37	31	4	2	
IV	13	11	1	1	
Differentiation					0.407
Well	39	26	7	6	
Moderate	58	35	15	8	
Poor	19	15	1	3	

^∗^ *P* values are based on chi-squared or Fisher's exact test. *P* < 0.05 indicates a significant association among the variables.

**Table 2:** The association between IL-18 and Ki-67 and cleaved caspase-3 expressions.

Tissue sample	IL-18 expression		*P* value	*r*
	Negative	Positive		
Ki-67				
Negative expression	21	22	0.003∗	-0.269
Positive expression	55	18		
Cleaved caspase-3				
Negative expression	42	15	0.07	0.169
Positive expression	34	25		

^∗^ *P* values are based on Spearman's correlation coefficient test. *P* < 0.05 indicates a significant association among the variables.

**Table 3:** Multivariate Cox proportional hazard models for overall survival.

Variable	Overall survival (OS)	
	HR (95% CI)	*P* value
T stage (T1+T2 vs. T3+T4)	1.689 (0.521–6.159)	0.235
N stage (N0 vs. N1+N2)	12.374 (1.506–48.180)	<0.001^∗^
AJCC stage (I+II vs. III+IV)	2.297 (0.689–5.982)	0.046^∗^
Differentiation (well vs. moderate+poor)	6.556 (2.254–10.842)	<0.001^∗^
IL-18 (high vs. low)	4.375 (1.279–8.220)	<0.001^∗^

HR: hazard ratio; CI: confidence interval; *P* values are based on likelihood ratio test; ^∗^*P* < 0.05 indicates significant difference.

## Data Availability

The data used to support the findings of this study are available from the corresponding author upon request.
